# Identification of an *EPC2-PHF1* fusion transcript in low-grade endometrial stromal sarcoma

**DOI:** 10.18632/oncotarget.24969

**Published:** 2018-04-10

**Authors:** Marta Brunetti, Ludmila Gorunova, Ben Davidson, Sverre Heim, Ioannis Panagopoulos, Francesca Micci

**Affiliations:** ^1^ Section for Cancer Cytogenetics, Institute for Cancer Genetics and Informatics, The Norwegian Radium Hospital, Oslo University Hospital, Oslo, Norway; ^2^ Department of Pathology, The Norwegian Radium Hospital, Oslo University Hospital, Oslo, Norway; ^3^ Institute of Clinical Medicine, Faculty of Medicine, University of Oslo, Oslo, Norway

**Keywords:** fusion gene, EPC2, PHF1, RNA sequencing, low-grade endometrial stromal sarcoma

## Abstract

Recurrent chromosomal translocations leading to gene fusion formation have been described in uterine sarcomas, including low-grade endometrial stromal sarcoma (LG-ESS). Involvement of the PHF1 gene in chromosomal rearrangements targeting band 6p21 has been found in LG-ESS with different partners from *JAZF1* mapping in 7p15, to *EPC1* from 10p11, *MEAF6* from 1p34, and *BRD8* from 5q31.

In the present study, RNA sequencing of a LG-ESS showed a novel recombination of *PHF1* with the Enhancer of Polycomb homolog 2 (*EPC2*). RT-PCR followed by Sanger sequencing and FISH analysis confirmed the *EPC2-PHF1* fusion transcript.

## INTRODUCTION

Recurrent chromosomal translocations leading to gene fusion formation have been described in uterine sarcomas [1, 2]. The discovery of such fusion transcripts provides not only fundamental knowledge about the pathogenetic mechanisms behind tumor formation and progression, but opens up for the fusions’ or fusion products’ use as potential molecular diagnostic markers and eventually as targets for smart drugs [3–5]. Many of the fusions identified are specific for distinct neoplastic entities.

The PHD finger protein-1 (*PHF1*) gene, mapping on chromosome band 6p21, was first identified as being rearranged in low-grade endometrial stromal sarcoma (LG-ESS) [6]. The gene is known to recombine with four partners through different chromosomal rearrangements within the same tumor type. It was found fused with *JAZF1* (from 7p15; through an unbalanced 6;7-translocation) [6], with *EPC1* (from 10p11; through a 6;10-translocation) [6], with *MEAF6* (from 1p34; through a 1;6-translocation) [7], and, the so far latest addition to the list, with *BRD8* (from 5q31, through a 5;6-rearrangement) [8]. The very same gene is also involved in the pathogenesis of non-ESS, non-endometrial stromal tumors (EST) such as cardiac ossifying sarcoma [9] as well as benign, atypical, and malignant ossifying fibromyxoid tumors (OFMT) [10–12]. In OFMT, *PHF1* has been shown to generate a fusion transcript not only with *EP400* (from 12q24) [13], but also with *MEAF6* and *EPC1* [10]. In cardiac ossifying sarcoma, a *JAZF1*-*PHF1* fusion was demonstrated [9].

**Figure 1:**
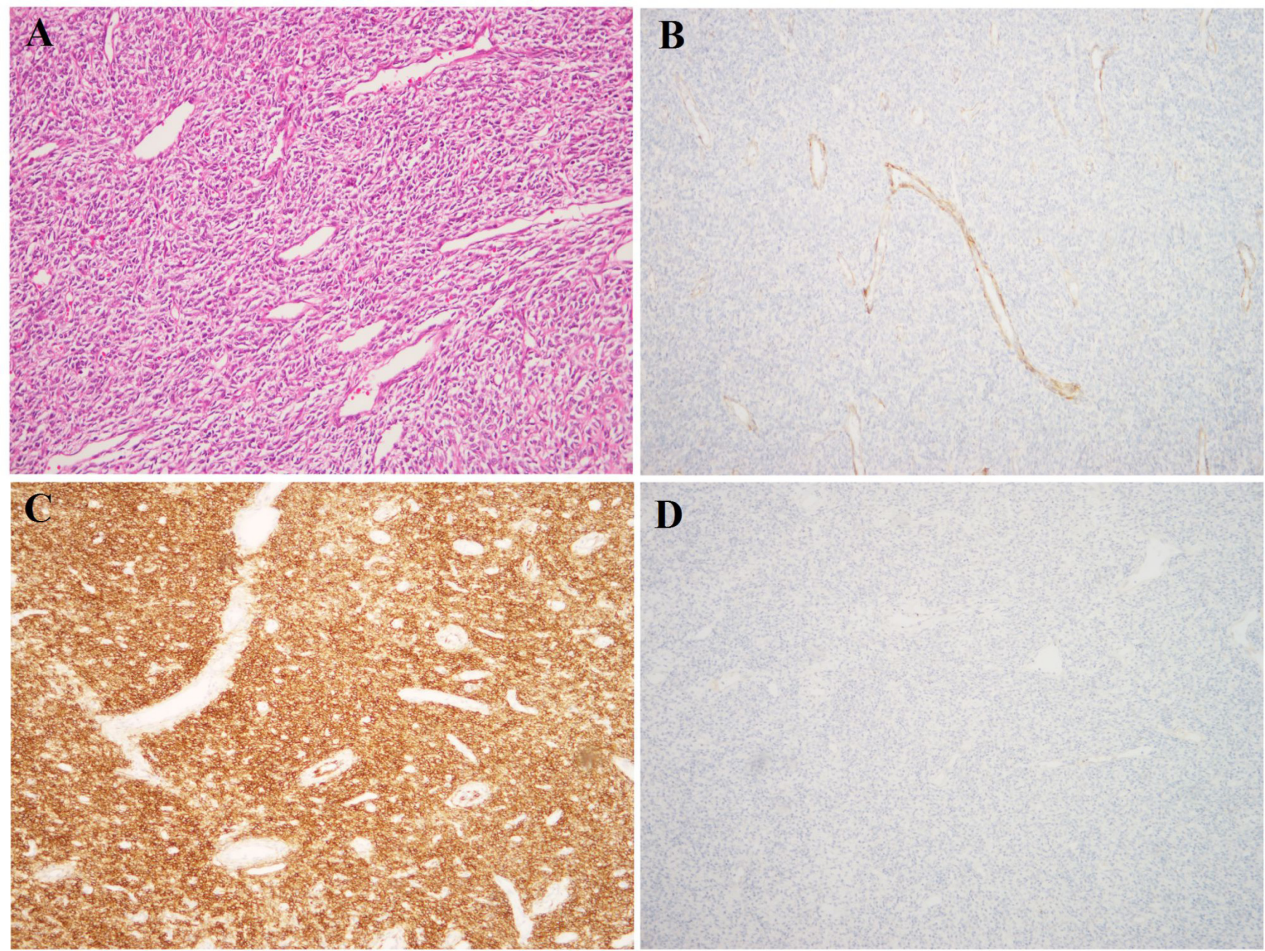
Histological examination of the LG-ESS (**A**) H&E – stained slide; tumor cells are spindle-shaped and monomorphic, with low-grade atypia. Multiple small thin-walled vessels are seen between tumor cells. The morphology is characteristic of LG-ESS; (**B**) Immunoexpression of caldesmon; (**C**) Immunoexpression of CD10; (**D**) Immunoexpression of desmin. The tumor has the characteristic IHC profile of LG-ESS, with expression of CD10 and absence of caldesmon and desmin.

We report here a new fusion partner for *PHF1* in an LG-ESS (Figure 1) detected by RNA sequencing and validated by RT-PCR followed by Sanger sequencing and Fluorescence *In Situ* Hybridization (FISH).

## RESULTS

**Figure 2:**
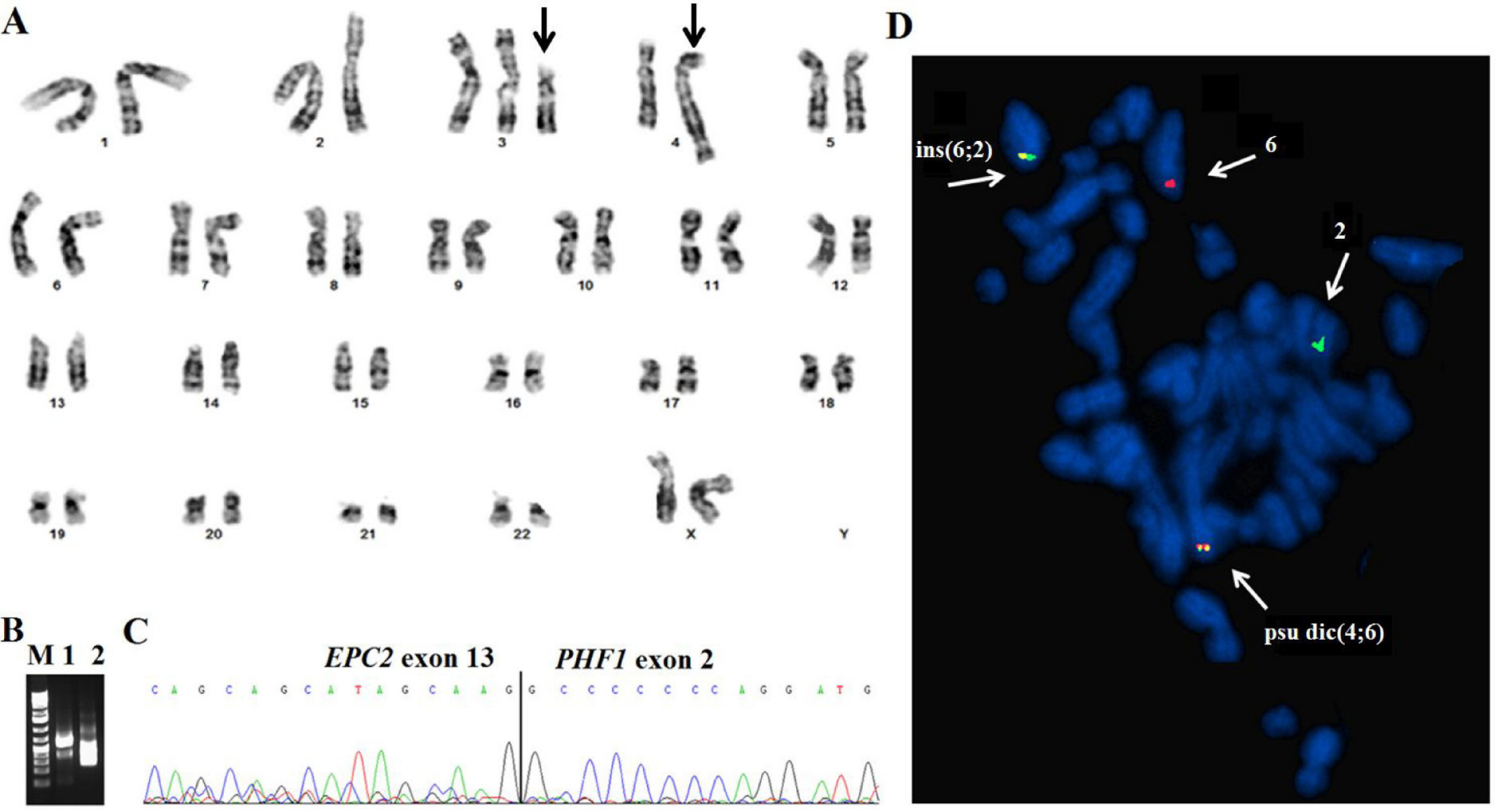
G-banding, RT-PCR, and FISH analysis of the low-grade endometrial stromal sarcoma (**A**) Karyogram of the LG-ESS. Derivative chromosomes, add(3p), and psu dic(4;6) are indicated by arrows. (**B**) Gel electrophoresis showing the amplified cDNA fragments. M, 1 Kb DNA ladder (GeneRuler, ThermoFisher); lane 1, amplification of cDNA fragment using the primers EPC2-2110F1 and PHF1-524R1; lane 2, Nested PCR using the primers EPC2-2266F2 and PHF1-376R1. (**C**) Partial sequence chromatogram of the amplified cDNA fragment showing the junction point of the *EPC2-PHF1* fusion. (**D**) Metaphase FISH for the detection of the *EPC2-PHF1* fusion gene. The green signal is the *EPC2* probe from 2q23 whereas the red signal corresponds to the *PHF1* gene from 6p21. The two fusion signals (yellow) were seen one on the psu dic(4;6) and ins(6;2).

The cytogenetic investigation of the LG-ESS showed an abnormal karyotype described as 47,XX,+add(3)(p11),add(4)(q35) [15] (Figure 2A). PCR investigations for ESS-specific fusion transcripts did not show the presence of any known fusions. A total of five chimeric transcripts were obtained using the FusionCatcher algoritm searching for novel fusions (Table 1). These were tested using the BLAT command (https://genome-euro.ucsc.edu/cgi-bin/hgBlat?command=start the program) to identify those with 100% identity in the genome according to the UCSC Genome Browser (update Dec. 2013, GRCh38/hg38). Only one out of five detected transcripts showed such identity, involving the Enhancer of Polycomb homolog 2 (*EPC2*) gene with *PHF1*.

**Table 1:** Fusion transcripts detected using FusionCatcher

5′-Chr	3′- Chr	5′- Partner gene	3′- Partner gene	Fusion sequence
17	17	*C17orf107*	*GP1BA*	GAAGAGATGGTGATAAAGACGCAGTTCCTCGTTCTTCCCCACACCCCTGC*CTGGAGCTGCAG AGGGGACGGCAAGTGACAGTGCCCCGGGCCTGGCTGCT
**2**	**6**	***EPC2***	***PHF1***	TGCCAAAGGTTACTCCCAGCAGTGCCATCAGCAGCATAGCAAG*GCCCCCCCAGGATGC **AATGGCGCAGCCCCCCCGGCTGAGCCGC**
2	2	*MBD5*	*EPC2*	TGAAGGCTTTATGGTTTACAGACAAGATCTTTAGAAGATAAGCACTAAAG*AGAGAACCACG AACCAGAAAGATTGGGCTTAAATGGAATAGCAGAGACAA
19	19	*EIF3K*	*ACTN4*	AGAAGAGAGCATTAAACCCAAGAACATTGTGGAGAAGATTGACTTTGACA*ACCTTCACGGC ATGGTGCAACTCCCACCTGCGGAAGGCAGGCACACAGAT
X	14	*MED12*	*IRF2BPL*	TCTTATAGCAGCAGCAGCAACAGCAACAGCAGCAGCAGCAGCAGCAGCAA*CAGCAGCAGC AGCAGCAGCAGCAGCAACAACAGCTCAACCACGTTGATGG

RT-PCR with specific primer combinations confirmed an in-frame fusion between exon 13 of *EPC2* (accession number NM_015630.3) and exon 2 of *PHF1* (accession number NM_002636.4) (Figure 2B and 2C; Table 2).

**Table 2:** Primers used for PCR and sanger sequencing analyses

Name	Sequence	Position	Gene	Accession number
*EPC2-2110F1* *PHF1-524R1* *EPC2-2266F2* *PHF1-376R1*	5′ -cagcttgtaaggacagttggc - 3′ 5′-ctcagagcgacagacacaac- 3′ 5′ -aatacacggacttcagcacc- 3′ 5′ -tatagcagcccatcagtcca- 3′	2110-2139 505-524 2266-2285 357-376	*EPC2* *PHF1* *EPC2* *PHF1*	NM_015630.3 NM_002636.4 NM_015630.3 NM_002636.4

Since the G-banding analysis did not show any rearrangements of 6p21, i.e., the band in which *PHF1* is located, we performed metaphase FISH experiments to see the chromosomal location of the *EPC2-PHF1* fusion. Two fusion signals (yellow color) were identified, one on the pseudo dicentric(4;6) and the other on the inserted(6;2) (Figure 2D). The revised karyotype incorporating the FISH and RNA-sequencing data thus became 47,XX, +add(3)(p11),psu dic(4;6)(q31;q15)ins(6;2)(p21;q23q23), +6, ins(6;2)(p21;q23q23).ish psu dic(4;6)(PHF1+, EPC2+), ins(6;2)(PHF1+, EPC2+;EPC2-)[4].

## DISCUSSION

Rearrangements of *PHF1* from chromosomal band 6p21 were first reported in ESS in 2006 when the gene was found recombined with two different partners, *JAZF1* (from 7p15) and *EPC1* (from 10p11), through unbalanced 6p;7p- and 6p;10p-rearrangements, respectively [6]. Subsequent cytogenetic and molecular studies detected additional partners for *PHF1*: *MEAF6*, recombined through a t(1;6)(p34;p21) [14] and, recently, *BRD8* from 5q31 [8]. We now report the fifth recombination partner, namely *EPC2* from 2q23.

The *EPC2* gene is a paralog of Enhancer of Polycomb homolog 1 of Drosophila (*EPC1*), a member of the Polycomb Group of Genes (PcG). *EPC2* is conserved from yeast to man. It is a component of an essential chromatin regulatory complex which has a potential oncogenic role as it contributes to cellular processes such as induction of apoptotic death [15]. Little is known about *EPC2* though different genetic studies suggest that deletion of its yeast homolog Ep11 [16] causes accumulation of cells in G_2_M, increases sensitivity to DNA damaging agents, leads to defective telomeric silencing, and may result in global loss of histone H2A as well as H4 acetylation. The gene also has an important role in homeotic gene silencing in Drosophila. Interestingly, a target knockdown screen of *EPC1* and *EPC2* in AML cell lines caused apoptosis and loss of stem cell potential [17]. Mutation of EP400 complex components *EPC1* and *EPC2* has been identified as pathogenetic events in AML [18].

The fusion of *EPC2* and *PHF1* led to a chimeric transcript retaining the entire coding regions from both genes interlocked in an open reading frame. The resulting putative protein would consist of 855 amino acid residues from EPC2 (AAH93818) and 662 amino acids from PHF1 (AAC52062.1); thus, the predicted protein sequence would consist of 1,517 amino acids in total.

The conserved domains from EPC2 include EPL1 (Enhancer of polycomb-like1) and –E-Pc-C (Enhancer of Polycomb C-terminus), complexes involved in transcriptional activation and heterocromatin formation, respectively. From the PHF1 protein are included its Tudor and PHD zinc finger as well as MTF2 domains, as was also the case in other *PHF1*-fusions previously described [6, 8, 14].

It appears that genes fused with *PHF1* through various translocations are involved in regulation of gene expression through formation of zing finger motifs or acetylation of histone proteins [14, 19]. They would therefore have the possibility of deregulating the transcription of a number of genes in LG-ESS as well as in seemingly unrelated tumors such as OFMT and cardiac ossifying sarcoma [9, 10]. This suggests a common neoplastic mechanism, namely rearrangement of the same gene, in these tumors that show little or no morphologic or immunophenotypic overlap. Indeed, the very same *EPC1-PHF1* and *MEAF6-PHF1* chimeric transcripts have been identified in both LG-ESS and OFMT [6, 12, 14], making these entities genocopies. In this regard, *PHF1* and the tumors characterized by its rearrangement do not differ principally from Soft Tissue Tumors in general where similar situations are known to be common [5].

As seen in the LG-ESS, OFMT, and cardiac ossifying sarcomas analyzed so far, the rearrangements of *PHF1* as the 3′- partner have breakpoints in exon 2 leading to retention of most of the gene sequence [6–10, 13, 14]. As a result of the rearrangements, the gene comes under the influence of a new promoter belonging to its 5′ partners. The fact that all detected alterations of *PHF1* are similar makes it likely that the gene is a key player in the tumorigenesis of these sarcoma subgroups [6, 10, 11]. Furthermore, the chromosomal aberrations behind the gene recombinations were always unbalanced allowing *PHF1* to retain its 5′-3′ orientation [6]. It is finally worthy of note that the rearrangement of 6p21, the chromosomal band where *PHF1* is located, was cryptic in the LG-ESS we report, hinting that it may be cytogenetically invisible also in other tumors. It seems certain that the use of FISH or molecular methods is necessary to detect this and related gene-level rearrangements in tumors with seemingly normal or complex karyotypes, perhaps especially when the lesions are histologically unusual.

The finding underscores the complex pathogenetic interplay that exists among ESS. Since *EPC2* is a paralogue of *EPC1*, one could speculate that the *EPC2-PHF1* fusion, too, will soon to be found in the rare OFMT and other non-ESS tumors.

## MATERIALS AND METHODS

### Case history

A 49-year-old woman presented with a tumor of the uterus whereupon total hysterectomy with left-sided salpingo-oophorectomy and right-sided salpingectomy was performed. Gross evaluation showed a 10 cm tumor with heterogeneous cut section in the uterine corpus, as well as several smaller nodules which were presumed to be leiomyomas. Morphological assessment of the large lesion showed a spindle cell tumor with low-grade atypia, 3 mitoses/10 HPF, but no necrosis. The histological diagnosis was LG-ESS. This was confirmed by immunostaining showing positivity for CD10, ER, PR, and SMA but negativity for desmin and caldesmon (Figure 1). The left ovary and both fallopian tubes were without tumor involvement as was the right ovary, which was also subsequently removed.

### G-banding and karyotyping

Fresh tissue from a representative area of the tumor was analyzed cytogenetically as part of our diagnostic routine [20]. The karyotype was written following the recommendations of the International System for Human Cytogenomic Nomenclature (ISCN) [21].

The study was approved by the Regional Committee for Medical and Health Research Ethics, South-East Norway (REK Sør-Øst; http://helseforskning.etikkom.no). Written informed consent was obtained from the patient. The consent included acceptance that the clinical details be published. The ethics committee’s approval included a review of the consent procedure. All patient information has been de-identified.

### Molecular genetic analyses

Total RNA was extracted from fresh frozen tumor tissue using miRNeasy (Qiagen, Hilden, Germany) and QIAcube (Qiagen). The RNA quality was evaluated using 2100 Bioanalyzer (Agilent, Santa Clara, California, USA) according to the manufacturer’s instructions. One µg of total RNA was reverse-transcribed in a 20 µL reaction volume using iScript Advanced cDNA synthesis Kit for RT-PCR according to the manufacturer’s instructions (Bio-Rad Laboratories, Oslo, Norway).

RT-PCR was used to investigate whether known ESS-specific fusion transcripts were present. The primers and PCR cycles are listed in previous publications [8, 22].

One µg of total RNA was sent for RNA-sequencing at the Genomics Core Facility, Oslo University Hospital and University of Oslo (http://oslo.genomics.no/). The sequencing was performed using an Illumina HiSeq 2000 instrument and the Illumina software pipeline. FusionCatcher (version 0.99.3a beta-April 15, 2014) with the associated ENSEMBL, UCSC, and RefSeq databases automatically downloaded by FusionCatcher (https://code.google.com/p/fusioncatcher/) were used for the discovery of fusion transcripts.

The primers used for PCR reactions and Sanger sequencing are listed in Table 2. The 25 μl PCR volume contained 12.5 μl Premix Ex Taq DNA Polymerase Hot Start Version (Takara Bio Europe/SAS, Saint-Germain-en-Laye, France), 1 μl of cDNA, and 1 µL of each of the forward and reverse primers. The primer combinations were EPC2-2110F1 and PHF1-524R1 for the first PCR reaction and EPC2-2266F2 and PHF1-376R1 for Nested PCR. The PCR amplifications were run on a C-1000 Thermal cycler (Bio-Rad Laboratories) with an initial denaturation at 94° C for 30 sec, followed by 35 cycles at 98° C for 7 sec, 55° C for 30 sec, 1 min at 72° C, and a final extension at 72° C for 5 min. Three µL of the PCR product were stained with GelRed (Biotium, Hayward, CA, USA), analyzed by electrophoresis through 1.0% agarose gel, and photographed. The remaining 22 µl PCR product were purified using the QIAquick PCR Purification Kit (Qiagen) and sequenced using 3500 Genetic Analyzer (Applied Biosystems). The BLAST (http://blast.ncbi.nlm.nih.gov/Blast.cgi) and BLAT (http://genome.ucsc.edu/cgi-bin/hgBlat) softwares were used for computer analysis of sequence data.

### Fluorescence *in situ* hybridization (FISH)

BAC probes were retrieved from the Human ‘32K’ BAC Re-Array library (BACPAC Resources, https://bacpacresources.org/). They were selected according to physical and genetic mapping data on chromosomes 2 and 6 (see below) as reported on the Human Genome Browser at the University of California, Santa Cruz website (May 2004, http://genome.ucsc.edu/). The clones used were RP11-899B15, mapping to 2q23 and containing the *EPC2* gene (labelled in green), and RP11-436J22 and RP11-600P03 mapping to 6p21 and overlapping with the *PHF1* locus (labelled in red). FISH was performed as described elsewhere [23]. Fluorescent signals were captured and analyzed using the CytoVision system (Leica Biosystems, Newcastle, UK).
